# Fibrillar tau promotes endothelial dysfunction through RAGE signaling and AGEs production

**DOI:** 10.1002/alz70855_106656

**Published:** 2025-12-24

**Authors:** Roberto A Guzman‐Hernandez, Silvia Fossati

**Affiliations:** ^1^ Temple University, Phialdelphia, PA, USA; ^2^ Alzheimer's Center at Temple, Lewis Katz School of Medicine, Philadelphia, PA, USA

## Abstract

**Background:**

Aggregated tau can spread from neurons or astrocytes throughout the brain, ultimately reaching the vasculature, possibly leading to cerebrovascular and neurovascular unit dysfunction. Currently, the mechanisms responsible for the effects of tau on endothelial cells (ECs) lining the vessel walls remain understudied, yet there is a growing interest in early vascular events preceding neurodegenerative pathology. We observed that protofibrillar tau mediates pro‐inflammatory EC activation and bioenergetic alterations, culminating in loss of barrier function. Here, we hypothesized that tau binding to the receptor for advanced glycation end‐products (RAGE) and the production of advanced glycation end products (AGEs)may play a role in the detrimental effect of tau in the EC barrier.

**Method:**

Human brain microvascular ECs (D3) were challenged with nM concentrations of protofibrillar 1N4R tau and co‐treated with FPS‐ZM1, a RAGE antagonist; or LR‐90, a scavenger for dicarbonyl intermediates responsible for AGEs. CRISPR/Cas9 RAGE KO ECs were treated with protofibrillar tau. Trans‐endothelial electrical resistance (TEER) was measured by the ECIS Zθ system as an *in‐vitro* model of the BBB. Cytokine production was assessed using an MSD V‐Plex Neuroinflammatory Panel 1. EC bioenergetics were measured by Seahorse Extracellular Flux Analyzer. Western blotting for VCAM‐1, RAGE, and tau was performed.

**Result:**

We showed reduced TEER following protofibrillar tau challenge, coupled with an increase in glycolysis, which correlated with a proinflammatory EC phenotype, ultimately rescued by modulating glycolytic metabolism. We observed that RAGE inhibition decreased entry of aggregated tau into ECs, reverting the pro‐inflammatory phenotype *in vitro*. Inhibition of tau‐mediated age production prevented the observed loss of barrier resistance and the increase in glycolysis. Further, RAGE deletion protected ECs from loss of barrier integrity and metabolic alterations.

**Conclusion:**

Our results suggest that fibrillar tau induces activation of RAGE signaling pathways, ultimately leading to loss of EC barrier function coupled with proinflammatory EC activation. Further, tau‐mediated AGE production may serve as an intermediate in this process, sustaining RAGE activation and metabolic alterations. Overall, our results provide insight into the mechanisms of tau‐mediated EC dysfunction.

